# Artificial Intelligence and Healthcare: A Journey through History, Present Innovations, and Future Possibilities

**DOI:** 10.3390/life14050557

**Published:** 2024-04-26

**Authors:** Rahim Hirani, Kaleb Noruzi, Hassan Khuram, Anum S. Hussaini, Esewi Iyobosa Aifuwa, Kencie E. Ely, Joshua M. Lewis, Ahmed E. Gabr, Abbas Smiley, Raj K. Tiwari, Mill Etienne

**Affiliations:** 1 School of Medicine, New York Medical College, 40 Sunshine Cottage Road, Valhalla, NY 10595, USA; rhirani2@student.nymc.edu (R.H.);; 2 Graduate School of Biomedical Sciences, New York Medical College, Valhalla, NY 10595, USA; 3 College of Medicine, Drexel University, Philadelphia, PA 19129, USA; 4 Department of Global Health and Population, Harvard T.H. Chan School of Public Health, Boston, MA 02115, USA; 5 Kirk Kerkorian School of Medicine, University of Nevada Las Vegas, Las Vegas, NV 89106, USA; 6 School of Medicine and Dentistry, University of Rochester, Rochester, NY 14642, USA; 7 Department of Neurology, New York Medical College, Valhalla, NY 10595, USA

**Keywords:** artificial intelligence, machine learning, telemedicine, chatbots, digital health, personalized medicine, neural networks, regulatory considerations

## Abstract

Artificial intelligence (AI) has emerged as a powerful tool in healthcare significantly impacting practices from diagnostics to treatment delivery and patient management. This article examines the progress of AI in healthcare, starting from the field’s inception in the 1960s to present-day innovative applications in areas such as precision medicine, robotic surgery, and drug development. In addition, the impact of the COVID-19 pandemic on the acceleration of the use of AI in technologies such as telemedicine and chatbots to enhance accessibility and improve medical education is also explored. Looking forward, the paper speculates on the promising future of AI in healthcare while critically addressing the ethical and societal considerations that accompany the integration of AI technologies. Furthermore, the potential to mitigate health disparities and the ethical implications surrounding data usage and patient privacy are discussed, emphasizing the need for evolving guidelines to govern AI’s application in healthcare.

## 1. Introduction

Artificial intelligence (AI) has emerged as an increasingly useful and reliable tool for various applications, particularly in healthcare. It has the potential to enhance the practice of physicians by facilitating improved efficiency and organization, thus improving patient care and outcomes. For patients, AI can improve access to care, which would likely lead to improved patient satisfaction and follow-up. However, like other technological advancements, AI has many limitations and potential pitfalls that must be thoroughly characterized and addressed before it can be trusted to be further integrated into healthcare. The importance of contextualizing this review broadly lies in understanding AI’s transformative potential in healthcare while acknowledging its limitations and ethical implications. In contrast to previous reviews, our focus extends beyond mere technological advancements to encompass a comprehensive examination of AI’s impact on healthcare delivery, patient outcomes, and societal implications.

The purpose of this review is to characterize the current state of AI use in healthcare starting from the field’s inception in the 1960s to present-day innovative applications in areas such as precision medicine, public health and immunization, medical education, and telemedicine. Furthermore, we emphasize the critical need to address social and ethical considerations associated with the expansion of AI usage in healthcare, particularly as they relate to health disparities. Lastly, the review will build from the identified limitations and considerations to provide guidance for effectively developing the next generation of AI in healthcare in a manner that promotes patient safety, accessibility, and inclusivity.

## 2. Section 1: Groundwork and Historical Evaluation

### 2.1. Artificial Intelligence: A Historical Perspective

AI is a broad term that encompasses an expansive landscape of research that attempts to model intelligent behavior without direct human involvement [1]. The very first question of AI dates to the 1950s with the “Turing Test”. Alan Turing posed a deceptively simple question: could a man-made device act and make decisions indistinguishable from those of humans [2]? This statement transformed AI from an amorphous concept to a well-defined goal for researchers and thinkers of the time to work towards. Turing posed the question, but many scholars credit the true conception of AI to the 1956 Summer Dartmouth Conference on AI. This conference drew the world’s leading data scientists, engineers, and mathematicians. They traveled to Dartmouth University to share ideas and collaborate with one another—all in the hope of laying the framework for practical applications of AI. Many of these experts stated that AI was indeed possible and, with keen foresight, claimed that AI would one day rival and surpass human intelligence [3,4]. 

The origins of industrial AI also date back to the 1950s and the primary goal of these primitive systems was for machines to emulate human decisions and actions [5]. The first robotic arm was developed in 1955 by General Motors [6]. Then, in 1964, Eliza, the world’s first chatterbot, was developed by Joseph Weizenbaum at the MIT AI Laboratory. Eliza’s system detected key words within the input text and then generated response based on reassembly rules. In this way, Eliza curated text responses that could simulate conversation with a human therapist [7]. Indeed, research in AI rapidly expanded in the 1960s, but many consider Shakey to be the crowning achievement of the decade. Shakey was the first robot that could interpret human instruction and then perform actions based upon those instructions. These contributions revolutionized the research landscape and proved that true AI was not simply a pipedream but a viable field of study with tangible results. 

AI with demonstrable medical applications began taking off in the 1970s [8]. INTERNIST-1, the world’s first artificial medical consultant, was created in 1971. The system utilized a search algorithm to arrive at clinical diagnoses based on patients’ symptoms. INTERNIST-1 represented a major shift in AI in clinical research because it had clear potential to take some of the onus of clinical diagnosis from healthcare providers and provided a mechanism for physicians to cross-check their differential diagnoses. By this point, it was so clear that AI had promising applications in medicine that the National Institutes of Health sponsored the very first AI in Medicine conference at Rutgers University [9]. The explosion of medical AI came, in part, from interdisciplinary meetings in which researchers in different aspects of AI shared both ideas and systems. One such system birthed from network integration was MYCIN. MYCIN was a system that used a set of input criteria to aid physicians in prescribing the correct antibiotics for patients diagnosed with infectious diseases [10]. The next major advancement took place in the 1980s at the University of Massachusetts. DXplain was a program that was designed to support clinicians in arriving at a medical diagnosis [11]. Clinicians could input symptoms and the system would return a potential diagnosis. The system was like INTERNIST-1 but expanded on the total number of clinical diagnoses that the system could derive and provided an early information bank for physicians to look for up to date medical information. 

The modern era of AI began in the early 2000s and saw some of the most expansive leaps for AI both in its applications to healthcare and also to human daily living. IBM created a question answering system in 2007 called Watson, which was able to outcompete top contestants and champions on the television show *Jeopardy*. This system used DeepQA, which used language processing to analyze data from different contexts and extract information from a wide array of sources to arrive at an answer [12,13]. This created opportunity for applications in the healthcare field, as inputs no longer needed to be symptoms and outputs could be more complex than purely clinical diagnosis. For example, in 2017, the Watson system was able to determine RNA binding proteins that were associated with amyotrophic lateral sclerosis [14]. New systems were made to support patient care in various capacities. Pharmbot, for example, was developed in 2015 to provide education regarding medication and treatment processes for patients and their families. 

### 2.2. Machine Learning and Neural Networks in Healthcare

**Figure 1:**
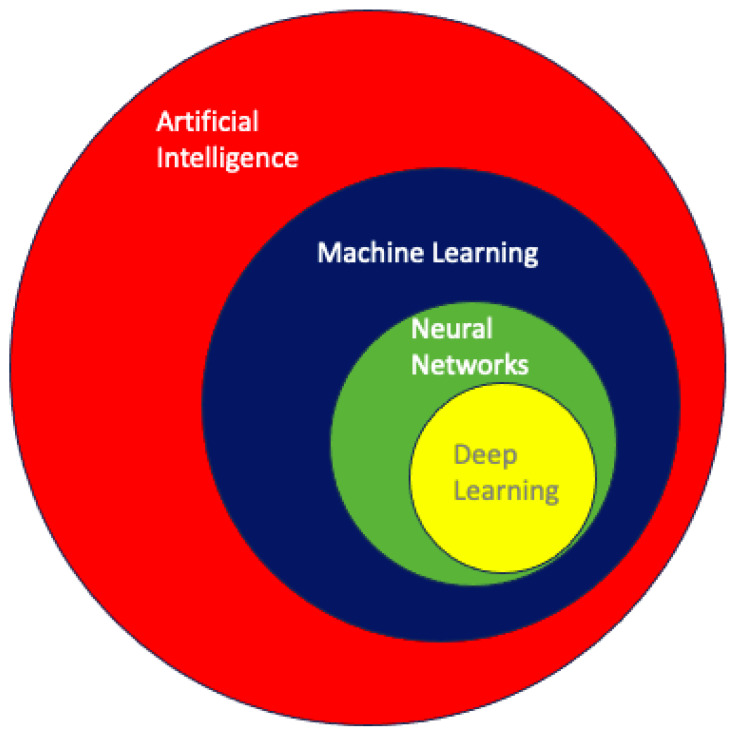
A concentric circle diagram demonstrating the relationships between different aspects of artificial intelligence [18]. Each circle delves into more granular aspects of the field, eventually converging on neural networks and deep learning—some of AI’s most sophisticated applications.

Within the broad and often difficult to navigate landscape of AI, machine learning (ML) is the process of using data and algorithms to make predictions. The goal of ML is to make these decisions purely through information gleaned from data rather than direct user input [15]. The specific types of algorithms used, therefore, are the primary focus of ML. ML algorithms are classically derived from mechanisms of statistical learning. Some common statistical learning methods include logistic regression, linear regression, and random forest (RF) [16]. K-nearest neighbor is a statistical learning mechanism that is used for data classification and regression [17]. These algorithms can be used in isolation or in rapid succession to assist in data processing, training, and task execution that are at the heart of ML as an entity. Figure 1 illustrates a concentric circle diagram delineating the hierarchical relationships within AI, progressively delving into more specific facets of the field. The diagram culminates in the innermost circles, emphasizing neural networks and deep learning as among the most sophisticated applications in AI.

Among the numerous frameworks of statistical inference that make up ML, we focus this discussion on deep learning and neural networks. Deep learning is a subset of ML that relies on mathematical models to emulate neuronal information processing. Artificial neural networks (ANNs) are generated to emulate the neuronal interconnections within the human brain. The nodes in ANNs are meant to represent neurons, receiving input from surrounding nodes in the network and sending output to other nodes [19]. This generates an interdigitating pathway that can allow information to be processed and relayed from node to node, much like the neuronal network that makes up the human central nervous system. Convolutional Neural Networks (CNNs) are a subclassification of ANNs that can both receive and send out multidimensional data. These networks can transmit more complex data and are useful for tasks like spatial recognition, audio processing, and language processing. These CNNs can, for example, use disease symptomology and clinical imaging to arrive at a clinical diagnosis, much like a trained physician would [20]. Research on CNNs surged in the 1970s, and the first neural networks were already put to clinical use by 1995 [21,22]. The later portion of this section will focus on clinical application of the various ML frameworks that we just described. Next, we review some of the most well-documented medical applications of ML: radiological image interpretation and dermatological pathology identification.

Radiological image interpretation is classically performed by highly trained physicians who use a methodical approach of comparing normal and abnormal imaging to arrive at clinical diagnosis [23]. Computer-Aided Detection (CAD) is an application of ML that assists clinicians and decreases the number of missed diagnoses when reviewing imaging studies [24]. These algorithms are designed to highlight potentially anomalous patterns on imaging to draw clinicians’ attention. Of note, these applications are not intended to replace the clinician, rather they are designed to aid the clinician with guided interpretation, thereby reducing the clinician’s false negative rate. Autonomous pathology detection without physician aid is also a rising application of ML. Since the 1970s, pixel analysis enabled ML algorithms to identify irregularities upon imaging studies, with the goal of detecting malignancy and metastatic disease [25]. Now, newer algorithms enable Magnetic Resonance Imaging (MRI) and ultrasound interpretation on par with that of practicing physicians [26]. Although diagnostic image interpretation is one of the many applications of ML in medicine, it provides meaningful evidence to support the overall value of ML in patient care. 

Lastly, we turn our attention to another major application of ML: the detection of skin pathology. There are multiple dermatological applications of ML including image-guided disease classification, pathology slide interpretation, assessment of skin disease using digital devices, and epidemiological studies [16]. Of note, skin cancer is by far the most common human malignancy, and thus, a great portion of the research is aimed at disease prevention and screening [27]. CNNs have been developed to recognize, detect, and classify dermatological images. These networks are trained using datasets containing thousands of normal and pathological images. For example, the GOOGLE Inception V3 was a CNN trained with over 1 million nonspecific images, and 129,450 dermatological and clinical images [16]. The CNN was able to detect dermatologic malignancy at levels on par with trained physicians [28]. In another study, the CNN was trained with 49,567 images and was able to accurately detect onychomycosis, a fungal infection of the nails, using only photos of the patients. The system achieved diagnostic accuracy that was superior to that of most dermatologists involved with the project [29]. 

**Figure 2:**
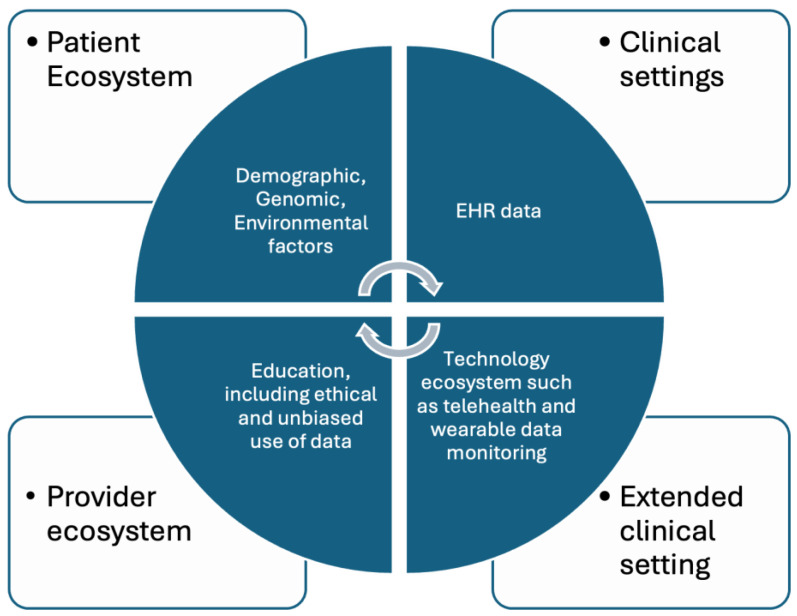
Application of AI in various domains of the healthcare ecosystem.

The applications of ML algorithms to radiological image processing and dermatology are only a few examples of the great storehouse of research conducted regarding medical applications of ML. AI has also been used for predicting patient risk for disease, triage and diagnostics, genetic engineering, as well as many more applications [30]. As research continues and AI becomes more integrated with the healthcare system, more applications are sure to arise, which are represented in Figure 2. With these advancements there may be even greater possibilities for improved patient care and more favorable outcomes—which are at the heart of medical research.

## 3. Section 2: Current Innovations and Applications

### 3.1. Advancing Personalized Medicine: Leveraging AI across Multifaceted Health Data Domains

In the traditional, one-size-fits-all approach of utilizing evidence-based medicine from high-quality experimental designs such as clinical trials, individual patient differences are often considered the undesired reality that adds complexity to the question being addressed. However, in precision medicine, this individuality is leveraged, and healthcare delivery is tailored to individuals’ unique health data such as genetics, lifestyle, and environment [31,32]. With the increasing adoption of wearables, improvements in next-generation sequencing, and increased implementation of electronic health records (EHRs), the health sphere has had a recent boom of individual healthcare data [32,33]. However, these personalized healthcare data are being collected at a rate leagues faster than they can be processed and analyzed, creating a bottleneck [34]. AI can address this bottleneck by increasing the quality and usability of these quickly growing data [35]. Various techniques can automate data cleaning, handling, and standardization of diverse data sources, which is crucial in making the data actionable. For example, Generative Adversarial Networks (GANs) and Autoencoders have shown promise in forecasting missing values and fixing tabulation mistakes in datasets [36]. The development of AI systems has paved the way for possible solutions to analyzing big datasets in a timely and cost-effective manner [37]. 

**Figure 3:**
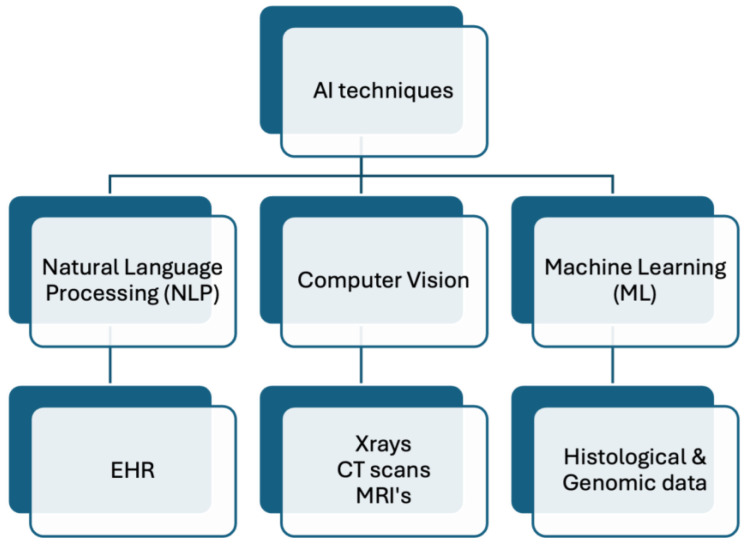
Non-exhaustive visual representation of AI techniques on example data types.

Multiple analysis techniques fall under the umbrella of AI, which employs various methods, algorithms, and data science approaches to analyze large datasets to extract meaningful information depending on the data type [38,39,40]. For example, ML techniques can be employed to analyze histological and genomics data, such as for classifying cancer [41]. Additionally, analysis of large amounts of health information stored as text data, such as EHR, might utilize Natural Language Processing (NLP) techniques [40,42], but analysis of medical imaging, such as CT scans, might utilize computer vision techniques [39]. This relationship is illustrated in Figure 3. It is important to note that these use cases are not rigid, and various techniques can be applied to the data to extract meaningful information. Examples of specific use cases along with AI analysis techniques are listed in Table 1. It is critical to ensure the quality of diverse data types when trying to leverage AI in healthcare. Advanced AI methods can play a key role in upgrading data accuracy and reliability across different data types, from EHR to medical imaging. As a result of reducing artifacts, errors, and noise, AI can improve the integrity and validity of personalized clinical treatment plans. As an example, deep learning models such as the Residual Encoder–Decoder Convolutional Neural Network (RED-CNN) employ CNNs and autoencoder architecture to remove noise from CT scans to improve the quality of the image itself, thereby guiding diagnosis and treatment planning [43]. This can be vital in conditions like COVID-19 when better imaging quality can help determine severity of infection and thereby changing management [35].

In genomics, AI algorithms can analyze sequences of DNA to identify genetic variations or mutations that may be linked to specific diseases or conditions. By recognizing patterns in these sequences, AI can help in diagnosing genetic disorders, predicting disease risks, or identifying potential therapeutic targets [41]. In proteomics, variations in protein types and quantities within an organism can result from different health conditions or responses to treatments. AI can identify these variations, offering the potential to uncover novel disease biomarkers or enhance our understanding of disease mechanisms. One group developed an AI model to aid with the diagnosis of thyroid malignancy from the proteomic samples of thyroid nodules with an estimated sensitivity of over 90% [49]. In addition to diagnostics, AI can also help in understanding which subsets of patients might benefit from a drug and which may not. One study was able to utilize AI models to detect which subset of gastric cancer patients would be sensitive to paclitaxel by analyzing their genome and uncovering a predictive biomarker [48]. 

The rise in EHR use globally provides vast amounts of data that can be leveraged to discover new insights [50]. AI algorithms are adept at analyzing EHRs, extracting patient histories, and uncovering trends that aid in clinical decision making and personalized treatment plans. For example, Fu et al. developed NLP algorithms using the Confusion Assessment Method (CAM) to detect delirium events from EHRs. These algorithms analyze patient charts for risk factors of delirium and, as a result, early interventions can be implemented to target high-risk patients [51]. 

The potential use cases of AI are not limited to biological aspects of disease, as AI can also be utilized to analyze psychological and sociological aspects of disease. In the realm of mental health care, AI-driven chatbots and virtual assistants represent a groundbreaking shift towards accessible and immediate support [52]. These AI applications extend their utility by aiding in behavioral intervention, offering users personalized strategies and exercises based on cognitive behavioral therapy principles. They augment traditional therapy by maintaining engagement between sessions through reminders and self-help tasks. The customization and adaptability of these chatbots, tailored to individual user needs and preferences, enhance user experience and engagement [52,53].

The use case extends beyond personal psychology and into sociological aspects of disease as well by tackling social determinants of health. In a study by Carroll et. al, an AI model was developed to analyze a wide array of data including patient demographics, medical histories, and, importantly, social determinants of health factors such as housing stability, access to transportation, and nutritional status. As a result, the model could predict a patient’s risk of hospital readmission by considering not just their medical history, but also factors like their living conditions and social support systems [54]. A patient identified as high risk due to factors like housing insecurity and limited access to nutritious food can be connected with social services for housing support and nutritional counseling, alongside their medical treatment. If the system picks up that the patient is in a financially precarious position, it can help recommend generic versions of medications or alert the healthcare team about prescription assistance programs that may exist to help address this need and improve adherence. In addition, if the patient is identified as having issues with access to transportation, then treatment modalities that require fewer in-person visits might be suggested such as larger proportions of telehealth visits.

AI’s potential not only comes from its ability to extract meaningful information from large datasets, but also through its ability to integrate findings from different sources [37,38,55]. The combination of molecular data such as genomics and proteomics with phenotypic information like patient history from EHRs plays a pivotal role in advancing personalized medicine [33]. The integration of individual health information, from the genomic level all the way to the sociological level, can allow for individualized treatment targeting each level. Utilizing AI to treat disease through this biopsychosocial lens allows for holistic comprehension and treatment of disease. 

### 3.2. Real-Time Monitoring of Immunization: Population-Centric Digital Innovations in Public Health and Research

Globally, an estimated 18 million children do not receive lifesaving vaccines, with an additional 25 million failing to complete the recommended vaccination schedule, as many communities in the low- and middle-income countries (LMICs) face barriers related to the accessibility of routine immunization services [56,57]. Lockdowns during the COVID-19 pandemic further disrupted the routine immunization services, worsening vaccination coverage and equity, affecting the vaccine demand in general and increasing the risk for secondary outbreaks of vaccine-preventable diseases [58,59,60]. With the increased accessibility of mobile phone technologies, digital health interventions have played a significant role in prioritizing immunization gaps by getting children caught up on their vaccinations and improving access to immunization programs to prevent outbreaks [56,61].

Alongside routine immunization, digitization has also improved the immunization information system (IIS) for COVID-19 vaccine programs through high-quality and comprehensive data capturing at the time of vaccine administration [62]. Studies highlight that 10–60% of immunization records have errors or lack important information, such as a patient’s demographics and lot numbers on vaccine vials, which is traditionally entered manually by the vaccine provider, including public health workers, nurses, or physicians [62]. However, in recent times, vaccine administration has become highly fragmented, with multiple providers administering vaccines, including pharmacists, workplace and school-based programs, travel medicine clinics, etc. [62,63,64]. Innovations in cloud systems and mobile technologies have opened new doors of possibilities to ensure the use of effective and comprehensive information systems to track vaccine uptake in complex immunization programs in upper-middle and high-income settings [62]. With tools like 2D barcoding, scanning of vaccination vials can help limit data slips and transcription errors by directly uploading the information into the IIS [62]. A similar approach can be used for streamlining the data entry procedures for demographic information and previous immunization records through scanning patient identification barcodes to extract all relevant information from the built-in electronic medical record software.

The use of digital health technologies including interactive dashboards and geographical information systems (GIS) has benefitted immunization campaigns by providing access to real-time information to assist policymakers and stakeholders to capture emerging outbreaks and enhance surveillance performances of eradication programs [65]. AI can also aid in reducing navigation barriers that influence immunization rates through features like immunization reminders and promotion campaigns [62]. In Africa, digital tools have supported polio eradication drives by leveraging the use of mobile phones to disseminate information for community mobilization and evaluating a campaign’s success through evaluations, through the use of GIS when producing maps for microplanning for better supervision and locating vaccination teams in northern Nigeria, and by the transmission of real-time data to support campaign monitoring [65].

Data dashboard systems also played an integral part in tracking COVID-19 cases and vaccine distributions, and have played a key role in strengthening equitable vaccine distribution, vaccination uptake, and preparedness for future disease outbreaks [66]. Soon after the World Health Organization (WHO) declared the global pandemic in early 2020, digital dashboards were created and made available to the public, such as the one created at Johns Hopkins University [66]. These digital dashboards used visual representations through translated data, positively influencing the general public’s understanding of health disease reporting and progression [67,68]. These advancements in digitalization of data reporting transformed health information systems during the pandemic [66]. Open-source web-based software have supported effective and precise reporting of pandemic indicators such as caseload and mitigation measurements, expanding beyond local or regional shadowing [66,69].

### 3.3. Revolutionizing Healthcare: The Synergy of Telemedicine and Artificial Intelligence

The term “telehealth”, as defined by the World Health Organization (WHO), refers to the delivery of healthcare services across distances through the use of information and communication technologies. This includes the exchange of valid information for the diagnosis, treatment, and prevention of diseases and injuries, as well as for research and evaluation, and the ongoing education of healthcare providers, with the overarching goal of advancing the health of individuals and communities [70]. In parallel, the Federation of State Medical Boards characterizes “telemedicine” as the practice of medicine facilitated by electronic communication, information technology, or other means between a physician in one location and a patient in another, with or without an intervening healthcare provider [71]. For the purposes of this manuscript, the term “telemedicine” will be employed as an umbrella term encompassing both telemedicine and telehealth. Over the decades, telemedicine has emerged as a crucial tool for delivering healthcare remotely [72]. This approach leverages a diverse array of technologies, including phone calls, video conferences, online health portals, mobile applications, and wearable devices [73,74,75,76]. Telemedicine applications can be broadly categorized into “real-time” and “store-and-forward” methodologies. “Real-time” applications involve active interaction between clinicians and patients through phone or video calls. On the other hand, “store-and-forward” applications entail the storage and transmission of data, such as images or vital sets, to clinicians for subsequent assessment and interpretation [73].

The utilization of telemedicine has significantly enhanced healthcare accessibility, particularly for individuals residing in rural areas with limited access to clinicians and those without means of transportation [73,77]. Moreover, telemedicine has demonstrated cost-effectiveness compared to traditional in-person visits, leading to reduced healthcare expenditures for patients [73,74,77]. The efficiency gains are further underscored by time savings for both patients and clinicians, as virtual appointments eliminate the need for travel and offer scheduling flexibility for physicians [73,77]. Beyond these advantages, virtual healthcare appointments mitigate unnecessary exposure to infections, a critical consideration amid the challenges posed by crowded waiting rooms in healthcare settings [78]. 

An additional consideration is the difficulty of travel for patients with physical disabilities who require a high level of coordinated services to get to a medical appointment. For some patients, like someone with advanced Parkinson’s disease [79], it can take an entire day to go back and forth to their medical appointment, which may only last about 15 min. This often requires multiple family members taking time off work as well as time spent waiting for transportation if the family does not have the appropriate vehicle to transport the patient. A telemedicine visit would relieve a great deal of strain on the many people tasked with helping this patient go to their appointment. Yet another challenge is transportation insecurity [80], which affects many patients in low-income communities. This can make it difficult for them to attend vitally important medical appointments, which could lead to worse outcomes. 

**Figure 4:**
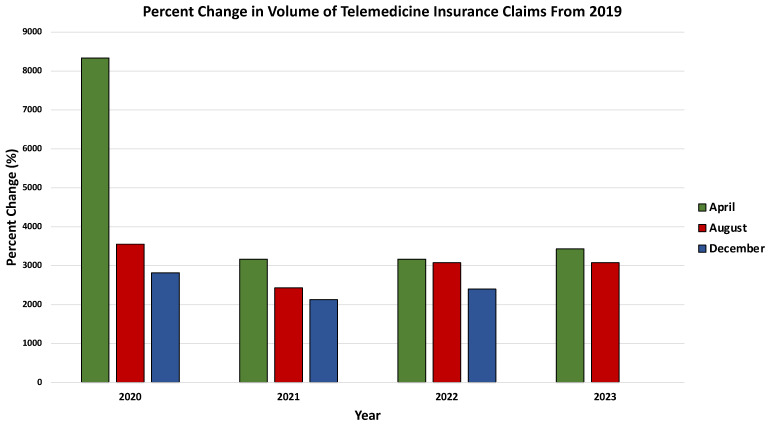
Visualization of the substantial surge in telemedicine usage during the COVID-19 pandemic and its sustained elevation post pandemic, presented through the percentage change in telemedicine health claims’ volume compared to the corresponding month in 2019. Data retrieved from: https://www.fairhealth.org/fh-trackers/telehealth (accessed on 17 November 2023).

**Figure 5:**
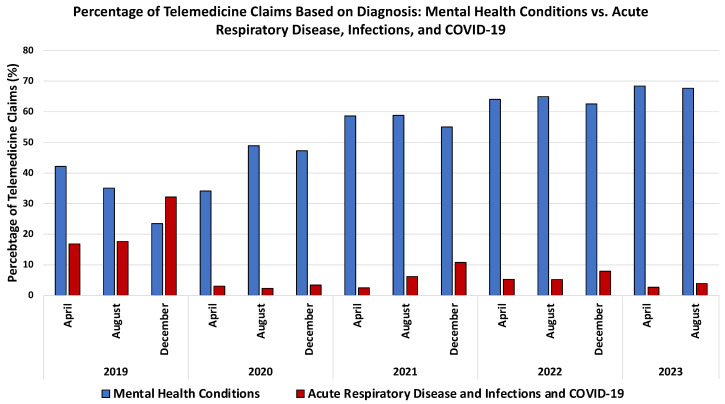
Comparative analysis of telemedicine claims highlighting the prevalence of behavioral and mental health conditions, a consistent trend observed during the pandemic and extending into both pre- and post-pandemic periods. Data retrieved from: https://www.fairhealth.org/fh-trackers/telehealth (accessed on 17 November 2023).

Notably, the onset of the COVID-19 pandemic triggered a substantial surge in telemedicine usage [78,81,82,83,84,85,86]. Many healthcare facilities swiftly transitioned from in-person to virtual visits during the early stages of the pandemic, ensuring the continued provision of care, especially for patients with chronic conditions [78,82]. This pivotal role of telemedicine in maintaining continuity of care was facilitated by various factors, including the rapid expansion of coverage by U.S. insurance companies for telemedicine visits, the allowance for clinicians to provide care across state borders, and the temporary relaxation of regulations by the U.S. Department of Health and Human Services (HHS) to facilitate telemedicine through personal devices and third-party applications without penalties under the Health Insurance Portability and Accountability Act (HIPAA) [82,87]. The significant increase in telemedicine usage during the pandemic is evidenced by data from the FAIR Health database, a nonprofit organization managing a vast repository of private health insurance and Medicare claims data in the US. Analysis of these data reveals a marked expansion in telemedicine usage during the pandemic and sustained elevation post pandemic, as depicted by the percent change in the telemedicine health claims’ volume compared to the corresponding month in 2019 (Figure 4). Notably, behavioral and mental health conditions emerged as the most common diagnoses associated with telemedicine claims during the pandemic, a trend consistent with pre- and post-pandemic periods (Figure 5) [85].

Telemedicine played a pivotal role in responding to and mitigating the spread of COVID-19 during the pandemic [78,82,88,89]. One such telemedicine service, known as “forward-triage”, was instrumental in managing the rising cases of infection. This service facilitated video consultations between clinicians and patients with suspected or confirmed COVID-19, enabling the evaluation of their condition to determine whether they required emergency department care or could continue remote management and isolation [78,88]. The implementation of “forward-triage” telemedicine not only reduced the unnecessary use of resources in emergency departments but also curtailed the unnecessary spread of the virus and minimized the exposure of healthcare providers [78,82,88]. Furthermore, telemedicine was employed for in-patient care to reduce nosocomial spread of COVID-19 and optimize the use of personal protective equipment (PPE). Providers conducted virtual rounds and communicated with patients using devices, demonstrating the versatility of telemedicine in diverse healthcare settings [89,90].

An integral facet of telemedicine, particularly evident post pandemic, is its significant role in mitigating physician burnout [91,92]. A survey encompassing 103 physicians at the Mayo Clinic revealed that 76% of respondents experienced heightened flexibility and control over patient care activities. Furthermore, approximately 30% reported an amelioration in burnout symptoms [92]. A similar trend was observed in a study by Chang et al., where physicians engaged in telemedicine exhibited lower burnout rates compared to their counterparts practicing traditional medicine [93]. Moreover, telemedicine has been linked to a comparatively lower incidence of medical malpractice claims than traditional in-person visits. It is noteworthy, however, that this disparity could stem from the relative novelty of telemedicine pre-COVID and the tendency of healthcare providers to employ it less frequently for serious medical issues [94].

The integration of AI has significantly enhanced telemedicine, with numerous studies highlighting its potential benefits [95,96,97,98,99,100]. AI’s ability to continuously update itself through learning from feedback and swiftly analyze data presents an opportunity to save considerable time and funds for healthcare providers and patients while also aiding clinicians in decision making [96,97,98]. Notably, AI algorithms have exhibited high sensitivity and specificity in interpreting medical images, rivaling the performance of healthcare professionals [95,101,102,103]. Approved by the Food and Drug Administration (FDA), these AI algorithms are proving instrumental in diagnosing conditions such as large vessel occlusion stroke [104,105], intracranial hemorrhage, pulmonary embolism, oncological lesions, seizures [46], acute abdominal abnormalities, breast cancer, tuberculosis, ophthalmologic diseases, skin lesions, and COVID-19, among others [101,102,103,106], and can lead to earlier intervention in a life-threatening situation like large vessel occlusion stroke.

Clinicians providing remote care through “store and forward” telemedicine stand to benefit significantly from AI algorithms, particularly in efficiently interpreting medical images and making accurate diagnoses. AI’s impact extends beyond image analysis to enhancing remote monitoring and management of patients’ conditions through smartphone software and wearable devices [96,97,99,107]. The existing capabilities of smartphones to support AI deep learning have led to applications designed to promote medication adherence and combat the spread of COVID-19 [96,99,107]. These applications use AI to remind patients to take medication and confirm its ingestion through video verification, reporting any discrepancies in real time to clinicians [96,107]. In response to the pandemic, AI-equipped smartphone applications were developed to remotely assess the likelihood of a patient being infected by analyzing their voice during speaking or coughing [99]. Smartwatches, incorporating AI algorithms, have been pivotal in monitoring vital signs and detecting conditions such as atrial fibrillation. During the COVID-19 pandemic, smartwatches were utilized to accurately monitor the activity of chronic stroke patients during remote rehabilitation exercises [96,97]. As we move forward, further research should explore leveraging the myriad sensors within smartphones and wearable devices, pairing them with AI to monitor physiological parameters such as vital signs [75,96]. Such advancements hold the promise of revolutionizing telemedicine and improving healthcare outcomes.

AI-driven chatbots have gained substantial popularity in recent years [108,109]. These chatbots have found application in telemedicine and exhibit considerable potential for further advancements [89,96,110,111]. During the COVID-19 pandemic, an AI chatbot was deployed for screening and triaging patients, alleviating the strain on manned triage hotlines overwhelmed by incoming calls. This chatbot effectively classified patients based on their symptoms, directing them to the hotline, home quarantine, a COVID-19 clinic, or the emergency department as deemed appropriate [89]. Additionally, a separate chatbot has been successfully employed to notify patients of the results of genetic sequencing, with a positive reception reported among patients [96]. In Indonesia, a health-focused chatbot underwent testing, addressing patient inquiries related to appointment scheduling, general health information, registration, diseases, and drugs [110]. Impressively, this chatbot demonstrated a high rate of accurate responses, reaching 93.1% [110]. Furthermore, similar chatbots have been specifically trained to assess whether a patient is afflicted with COVID-19 or heart disease [111]. A comprehensive study evaluated the diagnostic capabilities of ChatGPT, a widely utilized chatbot in medical contexts [108]. The study focused on ChatGPT’s proficiency in providing intelligent medical diagnoses based on presented symptoms. While the findings indicated that ChatGPT holds promise in offering potential diagnoses and medical information, limitations were observed in terms of consistency in delivering accurate diagnoses and providing in-depth medical advice [112]. These results underscore the importance of integrating medical chatbots with the expertise of clinicians. It is imperative to recognize that, while medical chatbots show promise, further research and refinement are essential to enhance their reliability and seamless integration into telemedicine practices.

Researchers have pioneered AI-based systems designed to diagnose specific diseases or establish a differential diagnosis by leveraging patient information, thereby facilitating clinicians in making accurate diagnoses and streamlining workflow [113,114,115,116,117,118,119,120,121,122]. Numerous studies have showcased the efficacy of AI algorithms in diagnosing various conditions, including suicidal ideation in pregnant women [114] and heart disease [115], by extracting information from electronic medical records (EMRs) with variable accuracy [113]. Furthermore, AI algorithms have demonstrated their diagnostic capabilities in Alzheimer’s Disease through the analysis of transcripts of spontaneous speech, autism spectrum disorder by evaluating behavioral features [116], acute appendicitis in children through blood analysis [117], and mood disorders based on cytokine profiles [118], and distinguishing between Parkinson’s and essential tremor using tremor data from wearable sensors, achieving accuracy rates ranging from 82% to 94.69% [113,119]. Additionally, AI has been instrumental in automating the classification of pathology reports [123], assigning ICD-10 codes to diagnoses in EMRs [121], and making broad diagnoses from clinical notes [120,122], with the accuracy ranging from 80% to 92% [113]. Another noteworthy AI system was developed to diagnose patients based on their symptoms and medical inquiries, achieving an accuracy rate of 84.9% [113]. These AI-based systems, designed for intelligent classification and diagnosis, exhibit promising results and significant potential for integration into telemedicine, thereby assisting clinicians in efficient and accurate diagnoses.

However, despite the advancements, further research is imperative to enhance the accuracy and safety of these systems, ensuring their definitive usefulness in clinical practice. Telemedicine has emerged as an indispensable component of healthcare, particularly highlighted during the COVID-19 pandemic. Yet, several challenges to the widespread use of AI-based telemedicine persist. One of these challenges is resistance from individuals unfamiliar with technology [77,98]. This issue will likely resolve over time as the population turns over. However, action that can be taken sooner includes designing simple, user-friendly interfaces and programming AI to clearly direct patients through instructions for using the technology. Another challenge is the lack of or inability to afford necessary technology in certain regions [77,78,98], which is compounded by insufficient political support and policy frameworks to facilitate increased access and ease of use [78]. This is a pervasive societal issue that limits the effectiveness of healthcare as a whole, let alone telemedicine and AI. Initiatives should be launched that aim to raise funds to provide necessary technology to underserved communities. These initiatives should also focus on designing AI-driven telemedicine platforms that are cost-effective and can function efficiently on basic technology that is more likely to be accessible to these communities. Privacy and security concerns, particularly with AI implementation, compose another challenge [96,98]. To combat these concerns, those that are designing AI to operate in the sphere of healthcare should focus intensively on safeguarding patient information and privacy. AI should include a built-in defense against data breeches and effective contingency plans for when this occurs. Any entity that hopes to employ AI in healthcare should make sure that the AI was designed with sufficient mechanisms for patient data protection. Lastly, usability issues related to device battery life, compatibility, and network coverage remain a challenge [75,98]. Mitigating issues stemming from battery life and network coverage may prove difficult, as these are reliant on device manufacturers and service providers. However, compatibility can be addressed by healthcare providers and corporations by requiring designers of AI-driven platforms to develop these platforms to be compatible with as many devices as possible in order to increase patient access. 

Incorporating AI into telemedicine is constrained by suboptimal accuracy and consistency of performance [98]. Therefore, it is essential that AI applications in telemedicine be supervised by trained clinicians to mitigate concerns and ensure the delivery of high-quality care. As the role of AI in telemedicine continues to expand, it is crucial to reiterate that AI should not replace healthcare professionals but rather complement their functions and assist in achieving the ultimate goal of effective patient care. Ongoing research efforts should focus on improving both telemedicine and AI to continually enhance our ability to care for all patients.

## 4. Section 3: AI in Healthcare Engagement and Education

### 4.1. Exploring the Impact of Chatbots on Patient Engagement, Mental Health Support, and Medical Communication

The COVID-19 pandemic underscored a significant gap in healthcare accessibility, prompting many healthcare systems to leverage AI chatbots for information dissemination and patient engagement [124,125]. Across 37 institutions spanning nine countries, the Watson Assistant AI conversational agent facilitated communication about COVID-19 and addressed patient queries, yielding over 6.8 million responses within a five month period, with interactions lasting two to three conversational turns on average [124]. In the post-COVID era, the utilization of chatbots in medicine has continued to surge [125].

One of the biggest implications of the utilization of these chatbots is that it results in higher patient engagement resulting in improved patient adherence to treatment because of shifting to patient-centric care [126]. Patients can ask these chatbots directly about any questions they might have at any hour of the day to explain common questions and even give recommendations to see a healthcare professional in person if appropriate given the context. Furthermore, these chatbots can be modified so that their explanations are appropriate to the literacy level the patient is comfortable with [127]. This is critical considering the abysmal rates of medical literacy in the United States which is a direct barrier to patient adherence [128].

Among these, Woebot has emerged as a noteworthy chatbot, incorporating cognitive behavioral therapy (CBT) into its algorithm [129]. In a study involving college students with self-identified depression, those utilizing Woebot experienced a notable decrease of 2.53 points in their Patient Health Questionnaire-9 (PHQ-9) scores, contrasting with no change observed in students provided only depression information [129]. Woebot’s empathetic conversational style and regular progress check-ins facilitated increased engagement and higher retention rates throughout the study [129]. The perceived empathy from the chatbot has proven pivotal in patients’ willingness to interact with the program [125,129,130]. Woebot also demonstrated efficacy in reducing drug cravings among substance abuse patients over a nine week period, along with improvements in depression and anxiety symptoms as measured by PHQ-8 and GAD-7 screenings [131]. Additionally, Woebot’s application in assisting new mothers experiencing postpartum depression led to a significant decrease in PHQ-9 scores compared to non-users [132]. Further research investigating the prior depression history of these patients is warranted to validate these findings. Notably, chatbots have effectively engaged special needs teenagers in managing their health, demonstrating increased self-care and sustained engagement [133].

For patients dealing with sensitive issues like substance abuse or depression, chatbots offer a convenient and stigma-free means of accessing support, especially for adolescents [131,134]. The concern arises, however, that the integration of chatbots into medicine may exacerbate health disparities for those without phone or computer access or those lacking tech proficiency. Navigating medical jargon proves challenging for many patients, as highlighted by an analysis of commonly asked questions revealing a predominant concern with radiology reports [135]. Chatbots aimed at simplifying complex radiology reports have shown varied effectiveness, with errors and missing information posing potential risks to patient well-being [136]. Hallucination, a process arising from assumptions made by the AI due to insufficient context, contributes to these errors. ChatGPT, like other chatbots, may also exhibit variability in responses, raising reliability concerns [136,137]. Despite these challenges, ChatGPT and Bard have demonstrated significant knowledge in radiology, lung cancer, neurosurgery, and ECG interpretation, with ChatGPT outperforming Bard in radiology board questions [137,138]. Both chatbots have been utilized in the education of radiology residents, showcasing their potential in enhancing medical training [139,140]. 

Beyond patient engagement, chatbots have been explored for their utility in triaging during mass casualty incidents, with Bard outperforming ChatGPT in accuracy [141]. The reasons for this difference warrant further investigation. Additionally, healthcare providers can leverage chatbots to impart educational information to patients, with ChatGPT excelling in providing understandable information on obstructive sleep apnea compared to Bard [142].

In summary, while chatbots offer promising avenues for improving healthcare communication and education, careful consideration and ongoing research are essential to address challenges and optimize their effectiveness in various healthcare contexts.

### 4.2. AI Integration in Medical Education: Transformative Trends and Challenges

AI has emerged as a potentially transformative force within the field of medical education, profoundly altering the methods by which students acquire knowledge, educators impart their wisdom, and healthcare professionals apply their expertise. In this section of the review article, we delve into the burgeoning integration of AI into medical education, encompassing its various applications in personalized learning, the introduction of AI-infused curricula in undergraduate medical programs, and the manifold challenges encountered in its implementation. The considerations surrounding the utilization of AI in medical education are two-fold. The first facet examines how educators can harness AI tools to enhance the pedagogical experience for medical students. The second dimension focuses on the imperative of incorporating a curriculum that equips students with the skills and knowledge to adeptly employ AI in their clinical practice upon completion of their medical training.

Despite its introduction in the 1950s, the integration of AI into medical education remained relatively stagnant until the early 2000s. Its adoption was cautious, and skepticism about its role persisted, contributing to its limited utilization in the field [143]. Numerous calls have emphasized the necessity of incorporating AI more prominently in medical education, particularly in response to its growing prominence in medical practice [144,145,146]. Advocates of AI suggest that it can serve as a bridge between traditional medical school curricula, which rely heavily on textbook-based learning and lectures, and modern teaching strategies that prioritize standardized content delivered through personalized video instruction, allowing students to progress at their own pace [147]. While there is a growing interest in AI’s application in medical education, comprehensive documentation of its implementation remains limited, with most information being anecdotal. Notably, AI’s most emphasized use in the classroom context is in personalized learning and its ability to offer specific feedback that might be otherwise challenging to provide due to time and faculty constraints [147].

Medical education places significant emphasis on the development of critical thinking skills [148]. Physicians are required to acquire, synthesize, and apply information effectively to make sound clinical decisions. This critical thinking process relies on a broad understanding of medical processes and pathologies, and the ability to integrate information from various sources to create well-informed treatment plans. While the pre-clinical phase of medical education primarily focuses on knowledge acquisition, medical institutions have long recognized the need to nurture critical thinking skills among students to facilitate their transition into the clinical phase [149,150,151]. The primary challenge in fostering critical thinking skills among pre-clinical students is the allocation of appropriate resources. In this context, researchers posit that AI can play a pivotal role. Instead of relying on one-on-one or small group interactions to assess and provide feedback on students’ critical evaluation of patient cases, AI offers the advantage of immediate and individualized feedback, allowing students to monitor their progress effectively [152,153].

Additional underexplored areas in the context of AI’s role in medical education include its potential to assist faculty in curricular development and assessment. In response to the dynamic nature of medical education, educators are increasingly challenged to deliver compelling and concise lectures that can effectively compete with the content available from external learning resources [154]. A recent article advocated the utilization of AI for evaluating existing curricula, positing that it could streamline the process of assessing effectiveness and student satisfaction [155]. With proper planning, AI can not only streamline the assessment process but also contribute to the creation of curricula that harmonize with external resources, fostering an engaging in-class learning environment. Given the scarcity of time among both faculty and students, AI-generated lecture materials hold the promise of striking a balance between providing distinctive learning experiences for students and minimizing the time commitments required for faculty to develop and implement such experiences.

A prominent theme within the literature is the imperative for AI technology to be integrated into the pre-clinical phase of medical education, similar to the teaching of biomedical sciences. Many experts contend that the mastery of AI is a skill that necessitates nurturing, akin to other competencies acquired during medical school [156,157]. Given the ubiquitous presence of AI in contemporary medical practice, the days of physicians opting out of its use in patient care are past. As a result, it has been proposed that AI, both in practical application and ethical considerations, should be incorporated into evidence-based medicine curricula as a complementary component [158,159]. Nevertheless, as with any paradigm shift, the integration of AI into medical education presents its share of challenges. The substantial volume of information that medical students are expected to grasp makes educators understandably cautious about introducing additional content into undergraduate curricula, particularly if it entails an increase in class time [160]. Teaching students how to effectively employ AI in their studies and future medical practice would not only demand additional time during the pre-clinical phase but also necessitate that faculty themselves feel sufficiently confident with the subject matter to instruct on it [160,161].

## 5. Section 4: Ethical Considerations, Limitations, and Future Directions

### Ethical and Societal Considerations in Integrating AI into Medical Practice

To integrate AI technology more extensively into medical practice, numerous ethical considerations must be thoroughly addressed. One prominent concern pertains to the handling of data, particularly patient information, as AI heavily relies on the analysis of preexisting data. A notable instance occurred in 2016 when the Royal Free London NHS Foundation Trust collaborated with DeepMind, providing patient information without affording patients autonomy over their data [162]. The acquisition of patient data by private entities, exemplified by Alphabet Inc.’s acquisition of DeepMind, poses additional risks amid the escalating frequency of healthcare-related data breaches [163]. Even deidentified patient data, compliant with HIPAA guidelines, may inadequately protect against reidentification through triangulation [164].

Furthermore, the implementation of AI in healthcare must address its potential influence on healthcare disparities. It will be important to ensure diversity in the people who are creating the AI algorithms, otherwise there will be an increased likelihood of having biases embedded in the algorithms leading to more health disparities [165]. A study by Obermeyer et al. revealed racial bias in a widely used commercial algorithm, indicating that Black patients were clinically sicker than their White counterparts for a given predicted risk score [166]. This bias stems from the definition and measurement of quality care and the utilization of data [164]. If not rectified, hidden flaws in quality measures may perpetuate healthcare disparities through AI models [167]. Additionally, the development of AI programs by researchers introduces the risk of bias, emphasizing the need to rectify preexisting disparities in data collection and quality care definition before advancing AI implementation. Furthermore, there is a great difference in the accuracy, sensitivity, and multimodality capabilities of various AI systems. For example, the progress made from GPT-3 to GPT-4 is a prime example of substantial advancements in AI’s capacity to comprehend and handle intricate data. To illustrate this point, GPT-4 was able to pass the bar exam and score within the top 10% of all test takers, while GPT-3.5 scored in the bottom 10%. The differences in outcomes are due to GPT-4’s ability to handle a broader range of data types including texts and images along with the benefit of being trained on 45 gigabytes of data compared to 17 gigabytes of data for GPT-3. However, this improvement in performance results in it being more expensive to implement GPT-4 compared to GPT-3 [168]. Therefore, it will also be important to ensure that when AI is used in underserved communities, that these communities are not relegated to cheaper, less effective forms of AI that can further perpetuate health disparities. Furthermore, it is crucial to consider that societies and individualistic cultures rapidly evolve, underscoring the need for AI programs to be updated with the help of experts in those particular societies to convey information in line with their evolving values. The relevant stakeholders in this case may be clinical leaders, social workers, case managers, medical ethicists, patient advocacy groups, and diversity, equity, and inclusion leaders [169]. Moreover, as the ethics of a society itself rapidly evolve, it is imperative to train AI to generate valuable outputs that align with contemporary ethical standards.

The safety of AI-driven healthcare is a critical ethical consideration. While studies demonstrate AI’s potential to enhance patient outcomes, issues persist, including a lack of standardization in reporting findings, limited comparison to current care practices, and the potential for unsafe recommendations [170,171,172,173]. The “AI chasm”, representing the gap between statistically sound algorithms and meaningful clinical applications, adds complexity to evaluating safety outcomes [174].

Establishing trust in AI-assisted healthcare systems is pivotal for ethical care delivery. Addressing concerns about data use, privacy, bias, and safety is crucial for fostering patient trust [164]. Robust privacy and security measures should be implemented to protect patient data in AI-driven healthcare systems. This can involve the use of encryption, access controls, and transparent compliance with regulations such as HIPAA. Transparency in AI healthcare is imperative, especially considering the “black box” nature of many algorithms. Failure to explain reasoning may undermine patient values, necessitating informed consent from patients before AI involvement in their care [175]. Ultimately, properly addressing these ethical considerations is essential to prevent harm, mitigate healthcare disparities, and foster trust between patients and the healthcare system.

Future directions in AI integration into healthcare should prioritize the development and implementation of standardized ethical frameworks and guidelines. Collaboration among stakeholders, including clinicians, researchers, ethicists, and policymakers, is essential to ensure that AI technologies align with ethical principles and patient-centered care. Additionally, ongoing research into improving AI algorithms’ fairness, transparency, and accountability is crucial to mitigate biases and ensure equitable healthcare delivery. Furthermore, investment in AI education and training for healthcare professionals will be instrumental in promoting responsible AI use and fostering trust among patients and providers. By addressing these challenges and advancing ethical AI practices, the healthcare industry can harness the full potential of AI to improve patient outcomes while upholding ethical standards and protecting patient privacy and autonomy.

## 6. Conclusions

To conclude, this manuscript underscores the transformative role of AI and telemedicine in reshaping healthcare. The integration of AI into telemedicine has significantly expanded its capabilities, with FDA-approved algorithms enhancing diagnostic accuracy, enabling remote monitoring through wearable devices, and contributing to efficient healthcare delivery. However, challenges such as accuracy, consistency, and ethical considerations necessitate careful supervision by healthcare professionals.

The manuscript further explores the impact of AI-driven chatbots on patient engagement and mental health support. While promising, concerns about reliability and potential exacerbation of health disparities underscore the need for ongoing research and refinement.

On the telemedicine front, the text delves into its pivotal role, especially during the COVID-19 pandemic. Telemedicine, encompassing real-time and store-and-forward methodologies, has become a crucial tool, enhancing accessibility, reducing healthcare expenditures, and mitigating unnecessary exposure to infections. The acceleration of telemedicine adoption during the pandemic highlights its indispensable contribution to maintaining care continuity, optimizing resource utilization, and improving overall healthcare accessibility.

Additionally, the manuscript illuminates AI’s role in advancing personalized medicine by leveraging diverse health data domains. From genomics and proteomics to electronic health records and sociological factors, AI’s ability to analyze vast datasets presents opportunities for tailored, holistic patient care. Ethical considerations, including data privacy and bias, must be addressed to ensure responsible AI integration.

In summary, the convergence of AI and telemedicine represents a formidable force in healthcare transformation. Ongoing research, collaboration between AI and healthcare professionals, and a cautious approach to ethical considerations are crucial for harnessing the full potential of these technologies. Together, they have the capacity to reshape healthcare delivery, improve patient outcomes, and pave the way for a more efficient, accessible, and patient-centered healthcare system.

While this manuscript highlights the transformative potential of AI and telemedicine in healthcare, it is important to acknowledge its limitations. One limitation is the rapidly evolving nature of both AI and telemedicine technologies, which may outpace the scope of this study. Additionally, the ethical considerations discussed are complex and evolving, requiring ongoing vigilance and adaptation. Furthermore, the impact of AI and telemedicine on specific patient populations, healthcare settings, and geographic regions may vary and warrant further investigation. Future research could delve deeper into these areas to provide a more comprehensive understanding of the challenges and opportunities associated with AI and telemedicine integration. Moreover, exploring the long-term effects of AI-driven interventions on patient outcomes and healthcare delivery models would be valuable for guiding future developments in this field.

## Figures and Tables

**Table 1:** Summary of AI in personalized medicine case studies.

Study/Case	AI Technique Used	Application Area	Key Findings	Implications for Personalized Medicine
Dermatologist-level classification of skin cancer [44]	Convolutional Neural Networks (CNNs)	Dermatology	Classification of skin lesions with a level of competence comparable to dermatologists	Enhances early diagnosis and treatment personalization along with increasing access in areas with low number of dermatologists
Patient-centered pain care using AI and mobile health tools [45]	Reinforcement Learning	Chronic pain	AI-driven cognitive behavior therapy (CBT) non-inferior to traditional CBT	Improves individualized treatment approaches and access to care
Improved referral process for specialized medical procedures like epilepsy surgery [46]	Natural Language Processing	Epilepsy and Neurological surgery	Detection of high-risk individuals who would benefit from epilepsy surgery	Ensuring timely and appropriate referrals for at-risk patients
Delineating ulcerative colitis from Crohn’s Disease [47]	Guided Image Filtering (GIF)	Gastrointestinal diseases	Improved diagnostic accuracy in complex presentation of inflammatory bowel disease	Enhanced diagnostic accuracy leading to targeted treatment strategies
Chemotherapy selection for gastric cancer [48]	Random Forest Machine Learning Model	Oncology	Able to predict which subset of patients would benefit from paclitaxel in gastric cancer	Development of predictive biomarkers to guide personalized drug treatment regiment

## Abbreviations

AI	Artificial intelligence
ML	Machine Learning
ANNs	Artificial Neural Networks
CNNs	Convolutional Neural Networks
CAD	Computer-Aided Detection
MRI	Magnetic Resonance Imaging
EHRs	Electronic Health Records
GANs	Generative Adversarial Networks
NLP	Natural Language Processing
CT	Computed Tomography
DNA	Deoxyribonucleic Acid
GIS	Geographical Information Systems
WHO	World Health Organization
HIPAA	Health Insurance Portability and Accountability Act
FDA	Food and Drug Administration
PPE	Personal Protective Equipment
EMRs	Electronic Medical Records
